# The Phytoene synthase gene family of apple (*Malus* x *domestica*) and its role in controlling fruit carotenoid content

**DOI:** 10.1186/s12870-015-0573-7

**Published:** 2015-07-28

**Authors:** Charles Ampomah-Dwamena, Nicky Driedonks, David Lewis, Maria Shumskaya, Xiuyin Chen, Eleanore T. Wurtzel, Richard V. Espley, Andrew C. Allan

**Affiliations:** The New Zealand Institute for Plant & Food Research Limited, Private Bag 92169, Auckland, 1142 New Zealand; Institute for Wetland and Water Research, Radboud University, Postbus 9010, 6500 GL Nijmegen, Netherlands; The New Zealand Institute for Plant & Food Research Limited, Private Bag 11600, Palmerston North, 4442 New Zealand; Department of Biological Sciences, Lehman College, The City University of New York, 250 Bedford Park Blvd. West, Bronx, New York, NY 10468 USA; The Graduate School and University Center-CUNY, 365 Fifth Ave, New York, NY 10016-4309 USA

**Keywords:** Apple, Carotenoids, Fruit skin, Fruit flesh, Phytoene, Phytoene synthase, Promoter, Transient activation

## Abstract

**Background:**

Carotenoid compounds play essential roles in plants such as protecting the photosynthetic apparatus and in hormone signalling. Coloured carotenoids provide yellow, orange and red colour to plant tissues, as well as offering nutritional benefit to humans and animals. The enzyme phytoene synthase (PSY) catalyses the first committed step of the carotenoid biosynthetic pathway and has been associated with control of pathway flux. We characterised four *PSY* genes found in the apple genome to further understand their involvement in fruit carotenoid accumulation.

**Results:**

The apple PSY gene family, containing six members, was predicted to have three functional members, PSY1, PSY2, and PSY4, based on translation of the predicted gene sequences and/or corresponding cDNAs. However, only PSY1 and PSY2 showed activity in a complementation assay. Protein localisation experiments revealed differential localization of the PSY proteins in chloroplasts; PSY1 and PSY2 localized to the thylakoid membranes, while PSY4 localized to plastoglobuli. Transcript levels in ‘Granny Smith’ and ‘Royal Gala’ apple cultivars showed *PSY2* was most highly expressed in fruit and other vegetative tissues. We tested the transient activation of the apple *PSY1* and *PSY2* promoters and identified potential and differential regulation by AP2/ERF transcription factors, which suggested that the *PSY* genes are controlled by different transcriptional mechanisms.

**Conclusion:**

The first committed carotenoid pathway step in apple is controlled by *MdPSY1* and *MdPSY2*, while *MdPSY4* play little or no role in this respect. This has implications for apple breeding programmes where carotenoid enhancement is a target and would allow co-segregation with phenotypes to be tested during the development of new cultivars.

**Electronic supplementary material:**

The online version of this article (doi:10.1186/s12870-015-0573-7) contains supplementary material, which is available to authorized users.

## Background

Carotenoid compounds have important roles in biochemical processes in plants such as light harvesting during photosynthesis and protecting the photosynthetic apparatus against damage. As secondary metabolites, these compounds accumulate in plant tissues to give attractive colours, which facilitate pollination and seed dispersal. In fruit and other plant tissues, colour is of high consumer and commercial value [1]. Carotenoids have potential health benefits in reducing the risk of diseases [2, 3]. In food crops, carotenogenesis contributes to nutritional quality through accumulation of alpha- and beta-carotene, which are major sources of pro-vitamin A [4–7]. Carotenoids serve as substrates for the biosynthesis of apocarotenoids such as abscisic acid and strigolactone which mediate stress and developmental signalling responses [8, 9].

Phytoene synthase (PSY) plays a pivotal role in the carotenoid pathway as the first committed step and acts to control flux through the pathway [10, 11]. The number of *PSY* genes differs between species, a result of duplication events, which have significance for function and modulation of carotenogenesis. Arabidopsis has a single *PSY* gene while two *PSY* genes have been reported for carrot [12, 13]. Tomato, cassava and members of the grass family, such as maize, rice and sorghum have three paralogs [14–17]. Since *PSY* plays such an important role in the carotenoid biosynthetic pathway, the implication of this gene duplication is not insignificant and may be related to producing carotenoids for diverse roles as suggested earlier [18, 19]. The presence of multiple PSYs in plants has resulted in distinct roles acquired by individual members [6]. In maize, *ZmPSY1* is important for carotenoid accumulation in endosperm as well as stress-induced carotenogenesis in green tissues. *ZmPSY2*, which is upregulated by light, is associated with photosynthesis [20]. *ZmPSY3* is stress-induced and specifically expressed in roots [21]. Similarly in rice, the multiple PSYs have distinct as well as overlapping roles. Rice *OsPSY1* and *OsPSY2* are involved in carotenoid biosynthesis under phytochrome control in green tissues, and as found in maize, *OsPSY3* was also up-regulated during stress treatments [22]. In tomato, virus-induced gene silencing (VIGS) of *SlPSY1* resulted in a yellow flesh phenotype, with complete disappearance of linear carotenoids and only trace amounts of other carotenoid compounds [15, 23]. On the other hand, silencing of *SlPSY2* and *SlPSY3* did not result in any obvious fruit phenotype, suggesting tomato *PSY1* has a dominant role in the fruit. This is probably because tomato *SlPSY2* functions mainly in chloroplast-containing tissues [24].

Apples are well known for their metabolites such as flavonoids and vitamin C, which are beneficial health compounds. Fruit flavour and colour are major apple breeding objectives because of their importance in determining consumer preferences [25–29]. In general, volatile apocarotenoids such as β-ionone and geranylacetone contribute to flavour while the accumulation of coloured carotenoid compounds in the fruit, together with chlorophyll and anthocyanins are responsible for fruit colour [30–32]. In a typical red skinned apple fruit, such as ‘Royal Gala’, the anthocyanin and carotenoid concentrations increase at maturity, while the chlorophyll concentration decreases [33]. We previously characterized the apple carotenoid pathway and found strong correlation between carotenoid accumulation in apple fruit and transcript levels of three genes, *zeta-carotene isomerase* (*Z-ISO*), *carotenoid isomerase* (*CRTISO*) and *lycopene epsilon cyclase* (*LCYE*) [34]. However, given the indispensable role *PSY* genes play in the carotenoid pathway as the first committed step, we have now also characterised the apple *PSYs* by assessing any overlapping or specialized roles they might have in fruit, from enzymatic function, protein localization and transcript levels. We also examined the transcriptional activation of the *PSY* promoters by APETALA2 domain/ethylene response transcription factors (AP2/ERF).

## Methods

### Sequence analysis

Apple PSY genes were identified in the phytozome database (http://phytozome.jgi.doe.gov). Multiple amino acid sequence alignments were performed with ClustalW [35] using default parameters and were manually adjusted in Geneious (www.geneious.com). Transit targeting peptides of full length PSYs were predicted using the ChloroP bioinformatic tool [36]. Phylogenetic analysis was conducted using MEGA6 [37]. Evolutionary relationships were inferred using the Neighbor-joining method, with 1000 Bootstrap re-sampling strategy. The database accession numbers of sequences used are: AtPSY (AAA32836), EjPSY1 (KF922363), EjPSY2A (KF922364), EjPSY3 (KF922367), MdPSY1 (KT189149 corresponds to MDP0000177623), MdPSY2 (KT189150 corresponds to MDP0000237124), MdPSY3 (KT189151 corresponds to MDP0000151924), MdPSY4 (KT189152 corresponds to MDP0000288336), MdSQS1 (AGS78117), MdSQS2 (AGS78118), MePSY1 (ACY42666), MePSY2 (ACY42670), MePSY3 (cassava4.1_033101m available at http://phytozome.jgi.doe.gov), OsPSY1 (AAS18307), OsPSY2 (AK073290), OsPSY3 (DQ356431), SbPSY1 (AY705389), SbPSY3 (AAW28997), SlPSY1 (ABM45873), SlPSY2 (ABU40771), SlPSY3 (Solyc01g005940), ZmPSY1 (Zea mays) AAX13806, ZmPSY2 (AAQ91837), ZmPSY3 (DQ356430).

### Plasmids and functional complementation

The pACCAR25ΔcrtB (ΔcrtB) plasmid has all the genes needed to produce zeaxanthin diglucoside, except for a gene encoding PSY [38]. The pACCRTE plasmid, carrying the bacterial crtE gene to produce geranylgeranyl pyrophosphate (GGPP), was constructed by removing the *crtY*, *crtI*, *crtB* genes from pAC-BETA [39] by digesting with *Sal*I followed by religation of the vector. The pAC-PHYT vector for producing phytoene in bacteria was used as a positive control [40]. To test functional complementation, fruit cDNA fragments of *MdPSY1, MdPSY2, MdPSY4*, without predicted transit peptides, were amplified and cloned into the pET200/D TOPO vector (Life Technologies, Carlsbad, California, USA) to give pETPSY1ΔTP, pETPSY2ΔTP and pETPSY4ΔTP constructs respectively. Competent cells of DH5α, carrying either the ΔcrtB or pACCRTE, were co-transformed with the *PSY* constructs. Transformed cells were selected on LB plates with 34 mg/L chloramphenicol and 50 mg/L kanamycin at 37 °C overnight. Fifty mL of Luria-Bertani (LB) broth was inoculated with one mL of overnight bacterial culture, supplemented with antibiotics and grown at 37 °C for 8 h before induction with 10 mM IPTG. This was followed by incubation at 28 °C in the dark, with shaking at 200 rpm for 48 h and then an additional 48 h without shaking. Cultures were centrifuged for 15 min in the dark at 4000 x g, resuspended with five mL of methanol containing 1 % butylated hydroxytoluene (BHT) and sonicated twice with 30s pulses on ice, at 50 % of output power using a Microson Ultrasonic Cell Disruptor XL2005 (Heat Systems, Farmingdale, New York) equipped with a tapered 3 mm microtip. Samples were centrifuged at 4000 x g to pellet disrupted cells and the supernatant flushed to dryness with nitrogen. The dried extract was resuspended with acetone for high-performance liquid chromatography (HPLC) analysis.

### Protein localization and fluorescent confocal microscopy

Full-length ‘Royal Gala’ apple fruit cDNAs were amplified by polymerase chain reaction (PCR) and cloned into a Gateway destination vector, pGWB441 [41, 42], in-frame with enhanced-yellow fluorescent protein under the Cauliflower Mosaic Virus 35S promoter. Transient expression of fluorescent fusion proteins in maize etiolated leaf protoplasts was visualized with a DMI6000B inverted confocal microscope with TCS SP5 system (Leica Microsystems CMS) as described previously [43]. Images were obtained by combining several confocal Z-planes.

### Carotenoid and chlorophyll extraction

A 50 mg dry weight (DW) sample of powdered freeze-dried material from each sample was moistened with water (approx 100 μl) and first extracted overnight in 2 mL of acetone:methanol (7:3) with 200 mg mL^−1^ CaCO_3._ Extracts were kept at room temperature and covered with foil to exclude light. The extract was centrifuged for 5 min at 21,000 x g, the supernatant removed and re-extracted with an additional 1 mL of acetone:methanol (7:3). This process was repeated 3 times. The combined supernatants for each sample were partitioned with equal volumes of diethyl ether and water, and the diethyl ether fraction removed. This process was repeated until the acetone aqueous phase was colourless. The combined diethyl ether fractions were dried under O_2_-free N_2_ and the carotenoids dissolved in 1 mL of 0.8 % BHT/acetone as previously described [44] and then analysed by HPLC.

### HPLC analysis

HPLC analysis was performed on a Dionex Ultimate 3000 solvent delivery system (Thermo Scientific, Sunnyvale, California) fitted with a YMC RP C30 column (5 μm, 250 x 4.6 mm), coupled to a 20 x 4.6 C30 guard column (YMC Inc. Wilmington, North Carolina) (column temperature 25 °C) and a Dionex 3000 PDA detector as previously published [34]. Phytoene was monitored at 280 nm and phytofluene at 350 nm. Coloured carotenoids and chlorophyll *b* were detected at 450 nm, while chlorophyll *a* and other chlorophyll derivatives were monitored at 430 nm. Carotenoid concentrations were determined as β-carotene equivalents /g DW of tissue. Chlorophyll *b* was determined using a chlorophyll *b* standard curve derived from a spinach extract [45]. Chlorophyll *a* and other chlorophyll derivatives were determined as chlorophyll *a* equivalents/g DW of tissue, again derived from a standard curve using the spinach extract and monitoring absorbance at 430 nm. β-carotene, and lutein were identified in the extracts by comparison of retention times and on-line spectral data with standards. All *trans*-β-carotene and lutein were purchased from Sigma Chemicals (St Louis, Missouri, U.S.A.). Other carotenoids were putatively identified by comparison with reported retention times and spectral data [46–50] and by comparison with carotenoids present in a spinach sample. Total carotenoid and chlorophyll content of the fruit tissue was also estimated using methods as previously described [51].

### RNA extraction and cDNA synthesis

Total RNA was extracted by tissue homogenisation in CTAB buffer using a modified method from one previously described [34, 52]. cDNA was synthesised from total RNA (0.5-1 μg) using Superscript III reverse transcriptase (Life Technologies, Carlsbad, California, USA) following the manufacturer’s protocol. Reaction components included 50 μM oligo dT (12) primer, 500 μM dNTPs, 1X reverse transcription buffer, 5 mM MgCl_2_, 10 mM DTT, 40 units of RNaseOUT and 200 units of reverse transcriptase. The reactions were incubated at 50 °C for 50 min.

### Quantitative real-time PCR analysis

Primers were designed using PRIMER3 software [53] to a stringent set of criteria. RT-qPCR was performed under conditions described previously [34, 54] (Additional file 1). First strand cDNA products were diluted 1:25 and used as templates for the PCR reaction. PCR analysis was performed using the LightCycler 1.5 system and the SYBR Green master mix (Roche, Penzberg, Germany), following the manufacturer’s protocol. Each reaction sample was analysed from biological replicates, with a negative control using water as template. PCR conditions were as follows: pre-incubation at 95 °C for 5 min followed by 40 cycles each consisting of 10 s at 95 °C, 10 s at 60 °C and 20 s at 72 °C. Amplification was followed by a melting curve analysis with continuous fluorescence measurement during the 65–95 °C melt. The relative expression was calculated using LightCycler software version 4 and the expression of each gene was normalised to apple *Actin* and *Elongation factor1-α* gene, whose expression are considered stable in these tissues [34, 55].

### Cloning of apple PSY promoters and apple AP2/ERF transcription factors

*MdPSY1* and *MdPSY2* promoter fragments (1.5 kb) were amplified from ‘Royal Gala’ genomic DNA using primer pairs PSYprom1F (ATTCACTTTCAGGGAGGCGAAC) and PSY1prom1R (GGTTTTGGGTCTTGAGTGTGAG), and PSY2promF (CAGTATCGCGAATTTTTCGT) and PSY2promR (GAGGGTGTGAGTATGTGAGCTG) respectively. PCR fragments were cloned into pGEM-T Easy vector (Promega, Madison, USA) and then sub-cloned as *Not *I fragment into pGreen II 0800-LUC vector, upstream of the Luciferase reporter [56].

The apple AP2/ERF transcription factors were cloned as previously described [56, 57]. cDNAs from expressed sequenced apple libraries [58] were cloned into pART27 binary vector using restriction enzymes or pHEX2 using Gateway cloning.

### Transient assay of promoter activation

Transient assays were performed as previously described [56, 57]. *Agrobacterium tumefaciens* strain GV3101 carrying a cloned *PSY* promoter construct or AP2/ERF construct were both resuspended in infiltration buffer (10 mM MgCl_2_, 0.5 μM acetosyringone) and infiltrated into the abaxial side of *Nicotiana benthamiana* leaves. The plants were left to grow for 2 days before 2 mm leaf discs were taken from infiltrated leaves and assayed with Victor 3 Multi-label Microplate Reader (Perkin Elmer, Waltham, Massachusetts, USA). Luciferase expression under *PSY* promoters relative to Renilla luciferase signals under the Cauliflower Mosaic Virus 35S promoter was measured.

## Results

### PSY sequence characterisation

Twelve apple PSY gene models were identified in the Phytozome sequence database (Additional file 1). Sequence analysis showed these gene models map to six positions on four chromosomes (3, 9, 11 and 17), suggesting six PSY genes are present in the apple genome [59]. We amplified four of these genes (*MdPSY1*-*4*) in fruit for analysis, while two of them, "*MdPSY5*" and "*MdPSY6*", which are present as additional genes on chromosomes 9 and 11 respectively, did not have transcripts available in the publicly available apple EST libraries [58] so were not further analysed.

In order to understand the roles of the multiple apple PSYs in carotenogenesis, we analysed their gene sequences. Comparisons of PCR amplified ‘Royal Gala’ cDNA, and genomic DNA fragments of these genes revealed a strong nucleotide and amino acid sequence similarity between *MdPSY1* on chromosome 17 and *MdPSY2* on chromosome 9, and between *MdPSY3* (on chromosome 3) and *MdPSY4* (on chromosome 11). *MdPSY1* has a predicted 400-amino acid protein while *MdPSY2* has a predicted protein of 401 amino acids. *MdPSY3* has a stop codon after residue 130, from the original ORF start site, resulting in a truncated protein. There is a potential methionine start site at residue 150, which could result in a 242-amino acid protein. However, it is possible this *MdPSY3* transcript, sequenced from four fruit cDNA clones, is a result of mis-splicing; when the *MdPSY3* transcript is compared with the *MdPSY3* and *MdPSY4* genomic DNA sequences, the stop codon appears to be the result of a 15 bp footprint sequence left on exon 3 during the splicing of intron 2. Thus, there could be other correctly spliced *MdPSY3* transcripts present in apple without the internal stop codon. Such correctly spliced transcripts would result in a protein sequence identical to *Md*PSY4, which has an ORF of 1158 bp, encoding a predicted 386-amino acid protein.

**Fig. 1:**
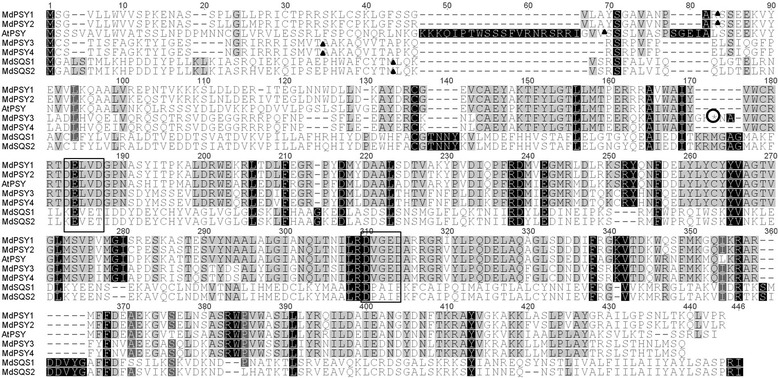
Alignment of the four apple Phytoene synthase (PSY) compared with Arabidopsis PSY and apple squalene synthase (MdSQS1 and MdSQS2). Multiple sequence alignment was conducted using ClustalW and manually adjusted in Geneious. The predicted chloroplast cleavage site by ChloroP is indicated by a black triangle. The stop codon present in PSY3 is indicated by a circled asterisk. Boxed sequence indicate the putative active site DXXXD [18]. Highlighting indicates similarity among residues ignoring the gaps in sequence; black, 100 %, dark grey >80 % and grey, >50 %

Multiple sequence comparisons of the predicted proteins indicated that *Md*PSY1 has 94 % identity to *Md*PSY2, while *Md*PSY3 has 98 % identity to PSY4 (Figure 1). In contrast both *Md*PSY1 and *Md*PSY2 have 54 % identity to *Md*PSY4. Comparing genomic and cDNA sequences revealed different exon-intron boundaries for the four *PSY* genes (Additional file 2). *MdPSY1* and *MdPSY2* have five exons and four introns, similar to the reported gene structure of the closely related loquat *EjPSY2A* [60]. *MdPSY1* and *MdPSY2* have similar exon sizes but differently spliced introns. *MdPSY4* has 6 exons and five introns, which is similar to *MdPSY3* (both in size and position). Compared with the *Md*PSY4 protein sequence, the stop codon in *MdPSY3* appears to be the result of a 15 bp footprint sequence left on exon 3 during the splicing of intron 2.

**Fig. 2:**
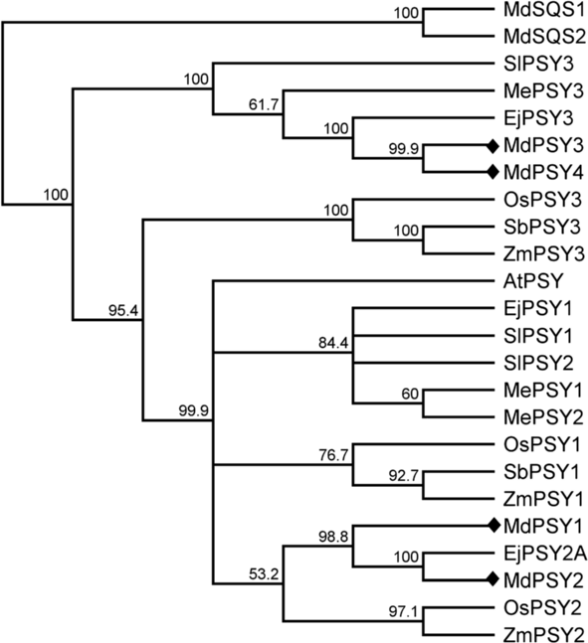
Phylogenetic tree was constructed using MEGA6 [37] from PSY and apple squalene synthase sequences retrieved from the GenBank database (except where noted): Arabidopsis, *At*PSY; loquat, *Ej*PSY1, *Ej*PSY2A, *Ej*PSY3; apple, *Md*PSY1, *Md*PSY2, *Md*PSY3, *Md*PSY4, *Md*SQS1, *Md*SQS2; cassava, *Me*PSY1, *Me*PSY2, *Me*PSY3; rice, *Os*PSY1, *Os*PSY2, *Os*PSY3; sorghum, *Sb*PSY1, *Sb*PSY3; tomato, *Sl*PSY1, *Sl*PSY2, *Sl*PSY3; maize, *Zm*PSY1, *Zm*PSY2, *Zm*PSY3. Evolutionary relationships were inferred using the Neighbor-joining method [85], with 1000 bootstrap re-sampling strategy. The four apple PSY sequences are indicated by diamond

Phylogenetic analysis of predicted amino acid sequences (including transit peptides) classified PSY1, PSY2, PSY3 and PSY4 into two distinct clades, supporting the observation that these pairs arose from a single duplication event (Fig. 2). Apple *Md*PSY1 and *Md*PSY2 form a clade with maize *Zm*PSY2, rice *Os*PSY2 and loquat *Ej*PSY2A. On the other hand, *Md*PSY3 and *Md*PSY4 grouped together with loquat *Ej*PSY3, cassava *Me*PSY3, and tomato *Sl*PSY3.

### Functional complementation

To test whether the apple *PSY* genes encode functional enzymes, we used a standard bacterial complementation method for assessing carotenoid pathway enzyme function [10, 61]. *Escherichia coli* test strains were produced by transforming them with either pACCRTE, which encodes the enzyme to produce GGPP in bacteria, or pACCAR25ΔcrtB (ΔcrtB) which requires PSY function to produce zeaxanthin and its glycosylated derivatives [38]. Next, vectors with cDNA fragments encoding the open reading frame of the apple *PSYs* with predicted transit peptides removed, were transformed into each of the test strains.

**Fig. 3:**
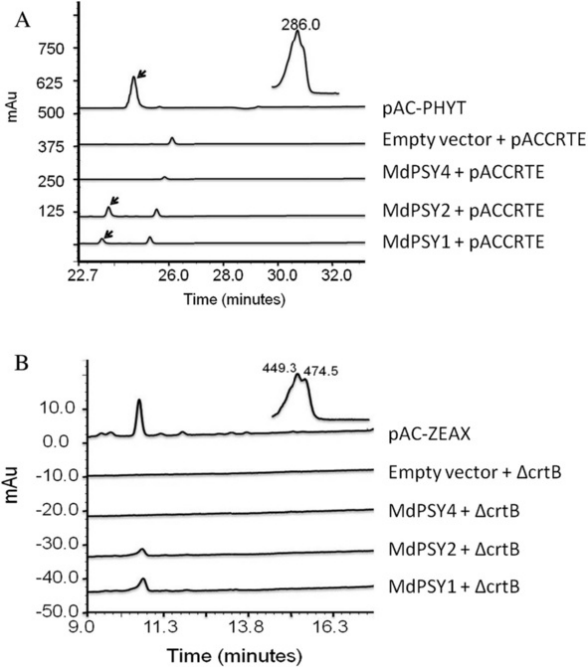
Functional complementation of apple PSY proteins. **a**. *Escherichia coli* cells harbouring the pACCRTE vector (which encodes CRTE, the enzyme catalyzing formation of geranyl geranyl pyrophosphate) were additionally transformed with apple PSY constructs or empty vector. Cells carrying the pAC-PHYT vector confer accumulation of phytoene [40] and were used as a positive control. HPLC chromatograms for the extracted pigments are shown. The peak representing phytoene (indicated by an arrow) was observed in cells with PSY1 and PSY2 constructs, but not with PSY4. The inset shows the absorption spectrum of the phytoene peak. **b**. *E. coli* cells harbouring pACCAR25ΔcrtB were transformed with the apple PSY constructs. Cells carrying the plasmid pAC-ZEAX [86] accumulating zeaxanthin were used as a positive control. The peak representing zeaxanthin diglucoside is shown. The inset shows the absorption spectra of the zeaxanthin diglucoside peak of pAC-ZEAX, which was similar to that from both PSY1 and PSY2 constructs with ΔcrtB

*MdPSY1* and *MdPSY2* constructs in cells with pACCRTE produced phytoene, with a peak whose retention time and spectral qualities were similar to the positive control (Fig. 3a). Similarly, ΔcrtB cells produced zeaxanthin diglucoside when transformed with *MdPSY1* and *MdPSY2* constructs (Fig. 3b). The extracts from these transformants had distinct yellow colouration and the peak retention times and fine spectral qualities were consistent with HPLC standards and previous publications (Fig. 3b) [22, 38]. Surprisingly, *Md*PSY4 in bacterial cells with pACCRTE or ΔcrtB did not result in the expected product peaks (Fig. 3). The same result was obtained with extended induction periods or using the full-length *Md*PSY4 including its transit peptide, suggesting *Md*PSY4 is not able to catalyse the conversion of GGPP to phytoene in bacteria.

### Protein localization

**Fig. 4:**
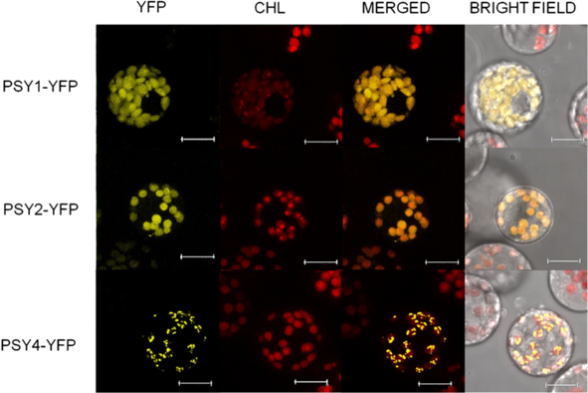
Transient expression of apple PSY-YFP fusion constructs in etiolated maize leaf protoplasts. PSY1 and PSY2 were localized throughout the plastids based on the fluorescent distribution pattern. PSY4 localized to speckles, suggesting localization to plastoglobuli [18]. CHL, chlorophyll autofluorescence; Bars = 10 μm

To ascertain the targeting of the apple PSYs, we fused PCR amplified fragments encoding apple PSYs including their predicted transit peptides to the enhanced yellow fluorescent protein (eYFP) [41, 42]. The fusion constructs were transiently expressed in maize etiolated leaf protoplasts and analysed using fluorescent confocal microscopy. Both *Md*PSY1 and *Md*PSY2 were co-localised with chlorophyll in the chloroplast confirming they are translocated to plastids (Fig. 4). *Md*PSY3 has a premature stop codon and was not further tested. *Md*PSY4 was localized to speckles associated with the chloroplasts (Fig. 4); these speckles are shown to be plastoglobuli using the maize plastoglobulin-2 as marker [18].

### Carotenoid accumulation in fruit

**Fig. 5:**
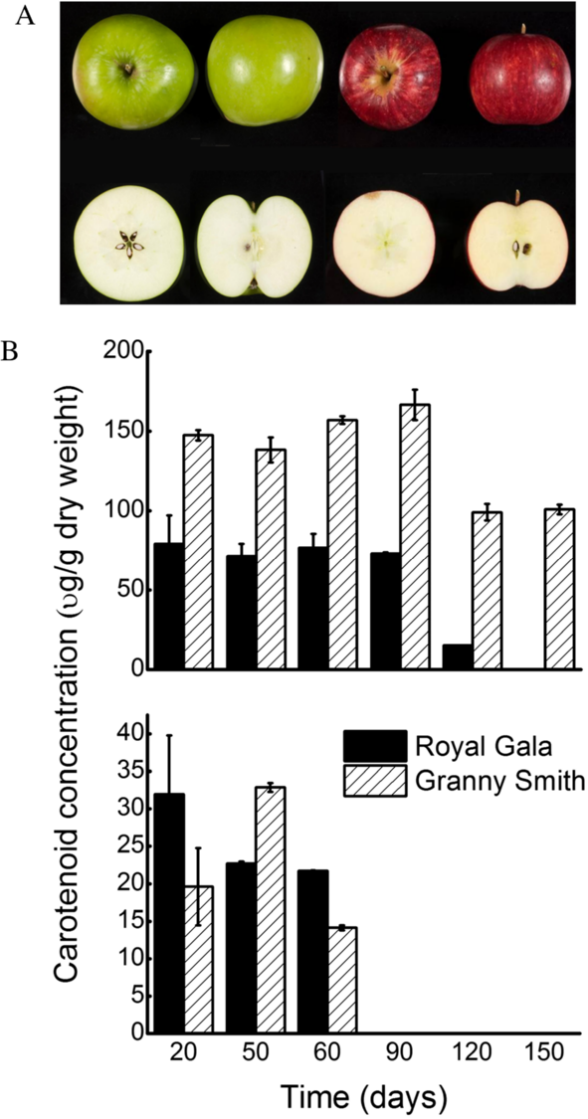
Carotenoid concentrations in fruit of apple cultivars selected based on the pigmentation of their skin and flesh. **a**. Ripe fruit (150 DAFB) of ‘Granny Smith’ (left) and ‘Royal Gala’ (right). **b**. Total carotenoid concentration as measured by HPLC in apple fruit skin (top panel) and flesh (lower panel). Fruit were harvested at different time points (days after full bloom) and separated into skin and flesh for carotenoid extraction and analysis. Error bars are standard errors from three biological replicates

To understand the role of *PSYs* in carotenogenesis during fruit development, we selected two commercial apple cultivars 'Granny Smith' and 'Royal Gala' based on the pigmentation of their fruit skin and flesh. ‘Granny Smith’ has a green skin and white flesh while 'Royal Gala’ has a red coloured skin with creamy flesh (Fig. 5a). Carotenoid and chlorophyll pigments were measured at different stages of fruit development. Total carotenoid concentration in ‘Granny Smith’ fruit skin was 2–5 fold greater than in ‘Royal Gala’ (Fig. 5b). In both cultivars, total carotenoid concentration in fruit skin appeared unchanged between 30 and 90 days after full bloom (DAFB) (~150 μg/g dry weight in ‘Granny Smith’ compared with ~75 μg/g dry weight for ‘Royal Gala’), followed by a significant decrease at 120 and 150 DAFB. Lutein and beta-carotene were the dominant compounds present in the analysed tissues and both compounds were up to 3-fold higher in ‘Granny Smith’ than ‘Royal Gala’ tissues (Additional file 3). The total carotenoid concentration in fruit flesh was about 7–10 fold lower than in skin and there was no clear pattern observed between the two cultivars. Total chlorophyll concentration (which includes breakdown compounds pheophytin a and b) in ‘Granny Smith’ tissues was 2–5 fold higher than in ‘Royal Gala’ (Additional file 3). A strong correlation was observed between total chlorophyll and carotenoid concentration (r = 0.97, p < 0.01) in these tissues, which suggested that most of the carotenoids present were associated with chloroplastic structures.

### Gene expression

**Fig. 6:**
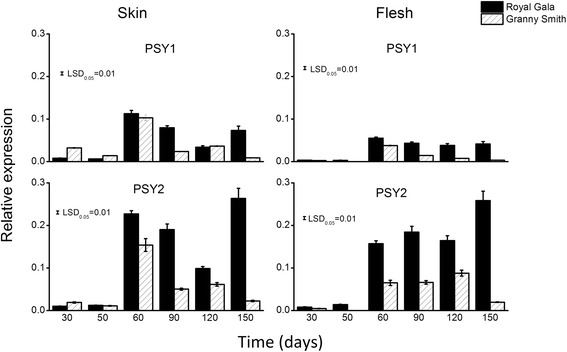
Gene expression profiles of *PSY* genes assessed in ‘Royal Gala’ and ‘Granny Smith’ apple fruit . *PSY* transcript levels in fruit skin and flesh picked at different time points (days after full bloom). The data were analysed using the target-reference ratios measured with LightCycler 480 software (Roche) using apple *Actin* and *Elongation factor1-α* (*EF1-α*) as reference genes. Data are analysed from biological replicates and presented as means ± SE (*n* = 4). Fisher's least significant difference (LSD) at *P* < 0.05 is shown

We analysed the transcript levels of *MdPSY1* and *MdPSY2* in fruit skin and flesh tissues of the two cultivars. Both *MdPSY1* and *MdPSY2* were, in general, higher (1.1 to 12 fold) in fruit skin than in flesh, consistent with higher carotenoid concentrations (2–5 fold) in fruit skin compared to the flesh [34]. Both *MdPSY1* and *MdPSY2* had similar tissue transcript profiles in both cultivars, though the transcript level of *MdPSY2* was higher than that of *PSY1* (Fig. 6). The transcript levels were reduced in young fruit skin and the highest transcript level was observed at 60 days after full bloom (DAFB). After this stage, transcripts decreased, with the exception of that in the ‘Royal Gala’ 150 DAFB tissue. In the flesh, *MdPSY1* and *MdPSY2* transcript levels were similarly reduced at the early fruit stages (30 and 50 DAFB) and increased at 60 DAFB. After this stage, *MdPSY1* transcripts reduced while *MdPSY2* transcript levels increased in ‘Royal Gala’ flesh until 150 DAFB.

**Fig. 7:**
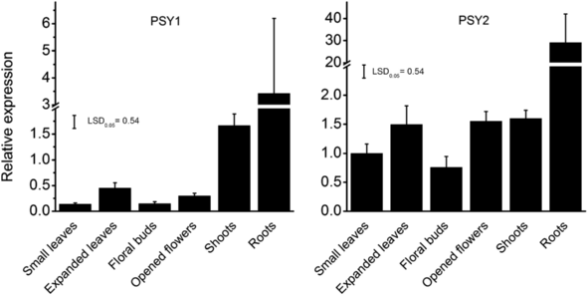
*PSY* transcript levels in different apple tissues from ‘Royal Gala’. Data were analyzed from biological replicates as described in Fig. 6 and presented as means ± SE (*n* = 4). Fisher’s least significant difference (LSD) at *P* < 0.05 is shown

*MdPSY* transcript levels were next examined in various apple tissues (Fig. 7). *MdPSY1* and *MdPSY2* were present in varying levels in all tissues examined, including both photosynthetic and non-photosynthetic. In small and expanded leaves as well as in open and unopened flowers, *MdPSY2* transcripts were at higher levels (3- to 5-fold) as compared to *PSY1.* Transcript levels for the two *PSYs* were similar in shoot tissues while in tissue-cultured roots, *MdPSY2* levels were about 9-fold higher than for *MdPSY1*. Taken together, *MdPSY2* represented the *PSY* gene with the most abundant transcripts. However, the *PSY* transcripts did not correlate with the total carotenoid levels in these tissues.

### Transient activation of *PSY* promoters

**Fig. 8:**
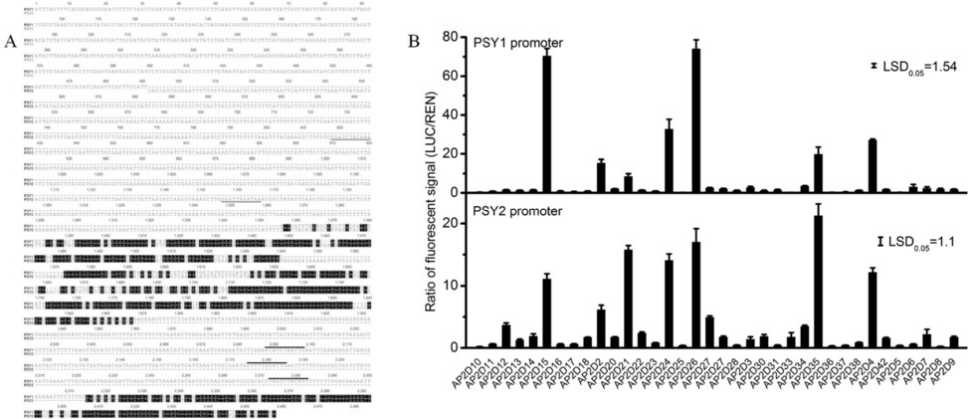
Transient activation of *PSY* promoters by apple AP2/ERF domain (AP2D) transcription factors. **a**. Alignment of ‘Royal Gala’ *PSY1* and *PSY2* promoters conducted using ClustalW in Geneious. Highlighting (black) indicate nucleotide similarity. RAP2.2 motifs are indicated by black (PSY1) and grey (PSY2) bars. **b**. Ratio of fluorescent signals measured from *Nicotiana benthamiana* leaves co-infiltrated with *Agrobacterium* constructs with *AP2/ERF* genes and a vector with firefly luciferase (LUC) under control of *PSY* promoter and Renilla luciferase (REN) under the control of CaMV 35S promoter. LUC/REN signal ratios were normalised to the basal promoter activity. Bars represent means ± SE (*n* = 4). Fisher's least significant difference (LSD) at *P* < 0.05 is shown

The transcript levels of *MdPSY2* compared with *MdPSY1* suggested these two paralogs are differentially regulated. To determine whether this differential regulation is related to diversity in the gene promoters, we amplified and sequenced 1.5 kb promoter fragments of *MdPSY1* and *MdPSY2* from ‘Royal Gala’ and found <30 % nucleotide similarity between these two gene promoters. Motif analysis by MatInspector [62] identified RAP2.2 (an APETALA2/ethylene response factor-type transcription factor) binding motifs in the *PSY* promoter sequences. Three RAP2.2 motifs were present in the *PSY1* promoter, all within 500 bp upstream of the ORF, while only two motifs were found in the *PSY2* promoter (Fig. 8a). In order to test functionality of the RAP2.2 binding motifs, both promoters were tested for transactivation with 36 apple AP2/ERFs transcription factors (TFs) [57] (Additional file 4, Additional file 6) using Agroinfiltration into young leaves of *Nicotiana benthamiana* [56, 63, 64]. Agrobacterium strains were separately transformed with constructs carrying PSY promoters cloned upstream of a Luciferase reporter or a gene encoding a AP2/ERF TF, which were together co-infiltrated into .*Nicotiana benthamiana* leaves. Transactivation was measured as an increase in luciferase levels compared to controls lacking a co-infiltrated transcription factor, normalised to 35S-Renilla [56]. The results showed differential trans-activation of the PSY promoters. For instance, the *MdPSY2* promoter was trans-activated (~15-fold increase) by AP2D21, a homolog of AtERF3, compared to a ~5-fold increase of *MdPSY1* promoter activity. Both *PSY* promoters were strongly activated by AP2D15 and AP2D26 homologs of AtRAP2.3 (47 % identity) and AtERF113 (49 % identity) respectively (Fig. 8b).

Physiologically relevant transactivation is predicated on overlapping *in vivo* expression of a candidate TF and the PSY target. Therefore, we analysed transcript levels of *AP2D15* and *AP2D26*, in the skin and flesh tissues of the apple cultivars (Additional file 5). Both *AP2D15* and *AP2D26* transcript levels increased from early fruit stage (30 DAFB) through to ripe fruit stage (150 DAFB) in both apple cultivars. Co-expression measured as correlation between gene expression of transcription factors and *PSYs* showed strong positive correlation between *AP2D26* and *PSY2* in fruit skin (Table 1), further suggesting a potential regulatory relationship.

**Table 1:** Pearson’s correlation between *AP2D15*, *AP2D26* and apple *PSY* transcript levels in skin and flesh of ‘Royal Gala’ (RG) and ‘Granny Smith’ (GS). Bold values are statistically significant at *P* < 0.05

				Transcription factors				
		*AP2D15*					*AP2D26*	
	RG		GS		RG		GS	
	skin	flesh	skin	flesh	skin	flesh	skin	flesh
*PSY1*	0.37	0.24	0.01	−0.22	0.45	0.28	0.5	−0.26
*PSY2*	0.56	0.56	0.61	−0.09	**0.81**	0.63	**0.81**	−0.1

## Discussion

Apples are consumed globally and are chosen to eat for their healthful metabolites and convenience. Consumers associate presence of nutritionally favourable compounds with fruit colour, partly contributed by carotenoids. Thus, carotenoid content has become an important apple breeding objective [25, 31, 34]. The phytoene synthase step is known to play a significant role in the carotenoid pathway because of its position as the first committed step, potentially controlling the flux downstream [65, 66]. Many plant species are known to have multiple *PSY* genes, including apple [34, 60].

The multiple *PSY* genes highlight the issue of functional diversity because of the potential to acquire a novel function, subfunctionalize or even lose the original function [67, 68]. The domesticated apple has 17 chromosomes, which may have been a result of both recent and older whole genome duplication (WGD) events [59]. The six apple *PSYs* are present on four chromosomes, which suggests that prior to the most recent genome duplication, at least two ancestral apple *PSY* genes on different chromosomes were present, resulting in the two homeologous pairs *PSY1*/*PSY2* and *PSY3*/*PSY4* described here.

*Md*PSY3/PSY4 cluster with maize and rice PSY3, which have had their function proven in bacteria. Others, such as cassava *Me*PSY3, tomato *Sl*PSY3and loquat *Ej*PSY3, which also share high homology to the apple PSY4 (66 %, 72 % and 96 % identity respectively) have not been tested or have been found to be non-functional [15, 21, 22, 60]. The non-functionality of *Md*PSY4 in bacteria could be because of the acquisition of a new function or perhaps mutation of some active sites. However, sequence analysis showed it was closer to PSY sequences than squalene synthase [18, 69]. It must be noted however, that while heterologous expression of plant genes in bacteria is a widely used method, the plastid environment where these enzymes function is absent, which may affect catalytic activity due to improper membrane localization of the enzyme and/or protein complex formation [19]. The localization of apple *Md*PSY4 to the plastoglobuli, in contrast to *Md*PSY1 and *Md*PSY2 in the chloroplast, may be important here in the sense that the catalytic activity of *Md*PSY4 could be influenced by its protein location. The tomato *SlPSY3*, for instance, was recently shown through virus induced gene silencing to affect carotenoid accumulation [15]. It remains to be seen if *Md*PSY4 has acquired a different function or its catalytic activity is affected by protein complex formation.

### *MdPSY2* has a dominant expression pattern in apple

We examined *MdPSY1* and *MdPSY2* transcript levels because of the ability of the encoded proteins to catalyze the conversion of GGPP to phytoene. Between these two genes, there was no tissue specific expression among the wide ranging apple tissues we examined to suggest subfunctionalization. Subfunctionalization of duplicated genes can take the form of complementary gene expression patterns in different tissues or partitioning protein function between paralogs [70, 71]. The absence of gene expression partitioning among the apple *PSYs* contrasts with what is observed in plants, such as maize, where subfunctionalization is observed among the *PSYs* [20]. This lack of difference in tissue-specific expression between *MdPSY1* and *MdPSY2* could be because the gene duplication between them is a recent event.

One obvious difference between *MdPSY1* and *MdPSY2* was their unequal gene expression levels. Unequal gene expression between paralogs in duplicated genomes can be an immediate consequence of the polyploidization or a result of changes introduced over time [72, 73]. The variation in gene expression between these *PSY* paralogs could be related to gene dosage effects or may simply be immaterial [74]. However, the higher relative expression of *MdPSY2* over *MdPSY1* is consistent with previous study where *MdPSY2* showed higher transcript levels (3–5 fold) over *MdPSY1* in different apple cultivars [34]. This could mean *MdPSY2* has a dominant role in apple and may be primarily responsible for this first carotenoid pathway step in apple tissues. However, both *PSY* transcripts do not correlate with total carotenoid levels, suggesting post-transcriptional processes may be important for determining flux through this enzymatic step. Recent knowledge on regulation of PSY in controlling carotenoid biosynthesis has shown that post-transcriptional mechanisms play a major role in the pathway [75]. With adequate expression of the *PSY* transcript, which we have shown is mainly *PSY1* and *PSY2* in apple fruit, it is then the stability of the protein which defines PSY as the rate limiting step in carotenogenesis [75]. In Arabidopsis this stability is modulated by the chaperone protein, AtOR [75]. Such knowledge has implications for apple breeding, where marker assisted selection is accelerating the breeding of better cultivars [76].

### Transactivation of *PSY* promoters

The possibility of different transcriptional mechanisms controlling the different expression of apple *MdPSY1* and *MdPSY2* is supported by the significant sequence differences in their promoters as well as the varied responses to transient activation by the AP2/ERF transcription factors we tested. The AP2/ERFs are implicated in many plant development processes such as floral development and response to biotic and abiotic stress [77, 78]. Their role in the carotenoid pathway was highlighted in Arabidopsis, when AtRAP2.2 was implicated in binding of the *PSY* promoter [79]. The apple AP2/ERF family has been characterised with some members implicated in fruit development [57, 58]. The expression pattern of *AP2D15* and *AP2D26*, with increased transcript levels as the fruit matured, suggests these genes have a role in fruit development. This, in addition to the transient activation of *PSY* promoters and the high correlation observed between their gene expression and *PSYs* point to a potential relationship where these AP2/ERFs are possibly regulating the *PSYs*. There is circumstantial evidence supporting *AP2/ERF*s in carotenogenesis in tomato, where they have been shown to regulate the carotenoid pathway negatively during fruit ripening. *SlAP2a* indirectly affects the carotenoid pathway through regulation of the ethylene pathway, as RNAi repression of this gene resulted in a concomitant increase in both ethylene and carotenoid compounds in fruit. Similarly, *SlERF6* was shown to affect carotenoid accumulation, where reduced expression by RNAi knockout resulted in increased carotenoid concentration in fruit. While these examples suggest both wild type genes are negative regulators of the pathway [80,81], they do not show any direct regulation of the carotenoid biosynthetic genes by the AP2/*ERFs*. The PSY step in carotenogenesis is under complex transcriptional and posttranscriptional regulation. The tomato *SlPSY1* and *SlPSY2* are targets of the MADS-box transcription factor Ripening Inhibitor, and *SlPSY1* gene expression and enzyme activity is inhibited by tomato Stay-green 1 [82, 83]. *AtPSY* is regulated by AtPIF1 through direct binding to the promoter to repress transcription and more recently, the Arabidopsis Orange (OR) and OR-like proteins are showed to be major posttranscriptional regulators of AtPSY, controlling AtPSY protein levels and carotenoid content [75, 84]. The further analysis of apple AP2/ERFs interactions with apple *PSY* promoters will increase our understanding of their role in the carotenoid pathway and maybe reveal how the apple *PSYs* are transcriptionally controlled.

## Conclusion

The data presented here suggest that the first committed carotenoid pathway step in apple is encoded by two functional genes *MdPSY1* and *MdPSY2*, with other apple *PSYs* playing little or no role in this respect. This has implications for apple breeding programmes that have fruit colour as a breeding target. Characterisation of the *PSY* gene family members increases our understanding of how the first carotenoid pathway step is controlled in apple and for instance, would allow co-segregation with fruit colour phenotypes to be tested during the development of new cultivars.

## Additional files

Additional file 1: List of primers used and apple PSY gene models (DOCX 14 kb) Additional file 2: The structure of the apple *PSY* genes. The exons (black bars) and introns (black lines) of the apple *PSY* genes were mapped by cDNA and genomic DNA sequence comparisons and constructed using FancyGENE version 1.4 (http://bio.ieo.eu/fancygene/). The location of the 15 bp insertion in *MdPSY3* is indicated by triangle. (JPEG 241 kb) Additional file 3: Table of carotenoid concentration (μg/g dry weight) measured by HPLC in skin and flesh tissues of ‘Royal Gala’ and ‘Granny Smith’ apple fruit at different stages of development. (DOCX 15 kb) Additional file 4: Phylogenetic tree of AP2/ERF amino acid sequences from apple and Arabidopsis using the Neighbor-joining method in MEGA6 and reliability of tree construction estimated by boostrap method based on 1000 replicates. Apple sequences used in this analysis are indicated with black triangles. The database accession numbers of the sequences used are presented in Additional file 6. (JPEG 60 kb) Additional file 5: Relative expression of *AP2D15* and *AP2D26* in ‘Royal Gala’ (RG) and ‘Granny Smith’ (GS) apple fruit as described in Fig. 6. Data are presented as means ± SE (*n* = 4). (JPEG 1012 kb) Additional file 6: List of genes and associated accession numbers used in the AP2/ERFs Phylogenetic tree (Additional file 4). (DOCX 13 kb)

## Abbreviations

bp: Basepairs
g: Gram
dw: Dry weight
DAFB: Days after full bloom
GGPP: Geranylgeranyl pyrophosphate
PSY: Phytoene synthase
