# Supplementary material for: The Phytoene synthase gene family of apple (Malus x domestica) and its role in controlling fruit carotenoid content
Source: BMC Plant Biol. 2015 Jul 28;15:185. doi: 10.1186/s12870-015-0573-7 (PMC4517366; doi:10.1186/s12870-015-0573-7)
Supplement: Additional file 1: — List of primers used and apple PSY gene models (DOCX 14 kb) [file 12870_2015_573_MOESM1_ESM.docx]

Additional file 1.

List of primers used

| **Primer name** | **Sequence** | **Purpose** |
| --- | --- | --- |
| PSY1ORF F | ATGTCAGGTGTTCTTCTCTGGGTG | Cloning cDNA ORF |
| PSY1ORF R | TCTAGGCACCAACTGCTTAGTTA | Cloning cDNA ORF |
| PSY2ORF F | ATGTCAGGTGTTCTACTCTGGGTG | Cloning cDNA ORF |
| PSY2ORF R | TCTAAGCACCAACTGCTTAGTTAA | Cloning cDNA ORF |
| PSY4ORF F | ATGTGTTCTACAATTTCCTTCGC | Cloning cDNA ORF |
| PSY4ORF R | TTGGGACATCAAAGTATGTGTTGATAG | Cloning cDNA ORF |
| PSY1gDNA F | CCTCACACTCAAGACCCAAAAC | Genomic DNA amplification |
| PSY1gDNA R | GAGATTGAGACCTCAAAATACCATC | Genomic DNA amplification |
| PSY2gDNA F | CTTGCCTAGTTTGACAAGGACCAT | Genomic DNA amplification |
| PSY2gDNA R | CTGACACATCTATCAACCACTCCA | Genomic DNA amplification |
| PSY3gDNA F | GATTTGGCTTGTCTTACTCGTGGG | Genomic DNA amplification |
| PSY3gDNA R | GTGAACGCTTTTAACTGCTTGAG | Genomic DNA amplification |
| PSY4gDNA F | CCGTGGGCATTTTCTGCAGTACAT | Genomic DNA amplification |
| PSY4gDNA R | TTGGGACATCAAAGTATGTGTTGATAG | Genomic DNA amplification |
| PSY1RT F | GAGAAGGTGTATGAAGTGGTGCTG | QPCR |
| PSY1RT R | TTCAAACCTTCAGTAATCCGTTCA | QPCR |
| PSY2RT F | GAGAAGATGCTAGGAGAGGAAGAGT | QPCR |
| PSY2RT R | CTTTGCCACTTGTCAGTCACCTTCC | QPCR |
| AP2D15RT F | GTGTGGTGGTGCTATCATTTCC | QPCR |
| AP2D15RT R | AGGAGGTCAGAGATGGTGTCAAG | QPCR |
| AP2D26RT F | CTGCCTCTACTCAACGAACACA | QPCR |
| AP2D26RT R | GCTGCGCGTACTGATAAAGATC | QPCR |

List of apple *PSY* gene models

| **Name** | **Accessions** | **Chromosome #** | **Location** |
| --- | --- | --- | --- |
| PSY1 | MDP0000177623 MDP0000251025 | 17 | 12M |
| PSY2 | MDP0000237124 | 9 | 11.8M |
| PSY3 | MDP0000151924 | 3 | 1.1M |
| PSY4 | MDP0000288336 MDP0000478884 | 11 | 0.59M |
| PSY5 | MDP0000321586 MDP0000261447 | 9 | 33M |
| PSY6 | MDP0000197515  MDP0000272976 MDP0000304577 MDP0000178953 | 11 | 0.56M |
