# Supplementary figures and images for: The Phytoene synthase gene family of apple (Malus x domestica) and its role in controlling fruit carotenoid content
Source: BMC Plant Biol. 2015 Jul 28;15:185. doi: 10.1186/s12870-015-0573-7 (PMC4517366; doi:10.1186/s12870-015-0573-7)

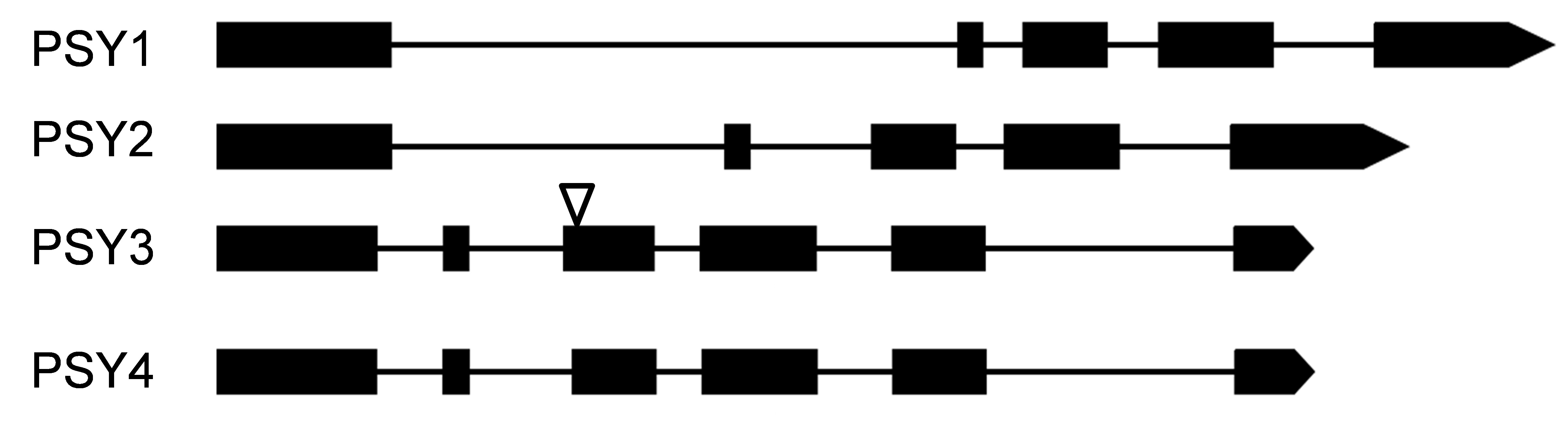

Supplement: Additional file 2: — The structure of the apple PSY genes. The exons (black bars) and introns (black lines) of the apple PSY genes were mapped by cDNA and genomic DNA sequence comparisons and constructed using FancyGENE version 1.4 (http://bio.ieo.eu/fancygene/). The location of the 15 bp insertion in MdPSY3 is indicated by triangle. (JPEG 241 kb) [file 12870_2015_573_MOESM2_ESM.jpg]

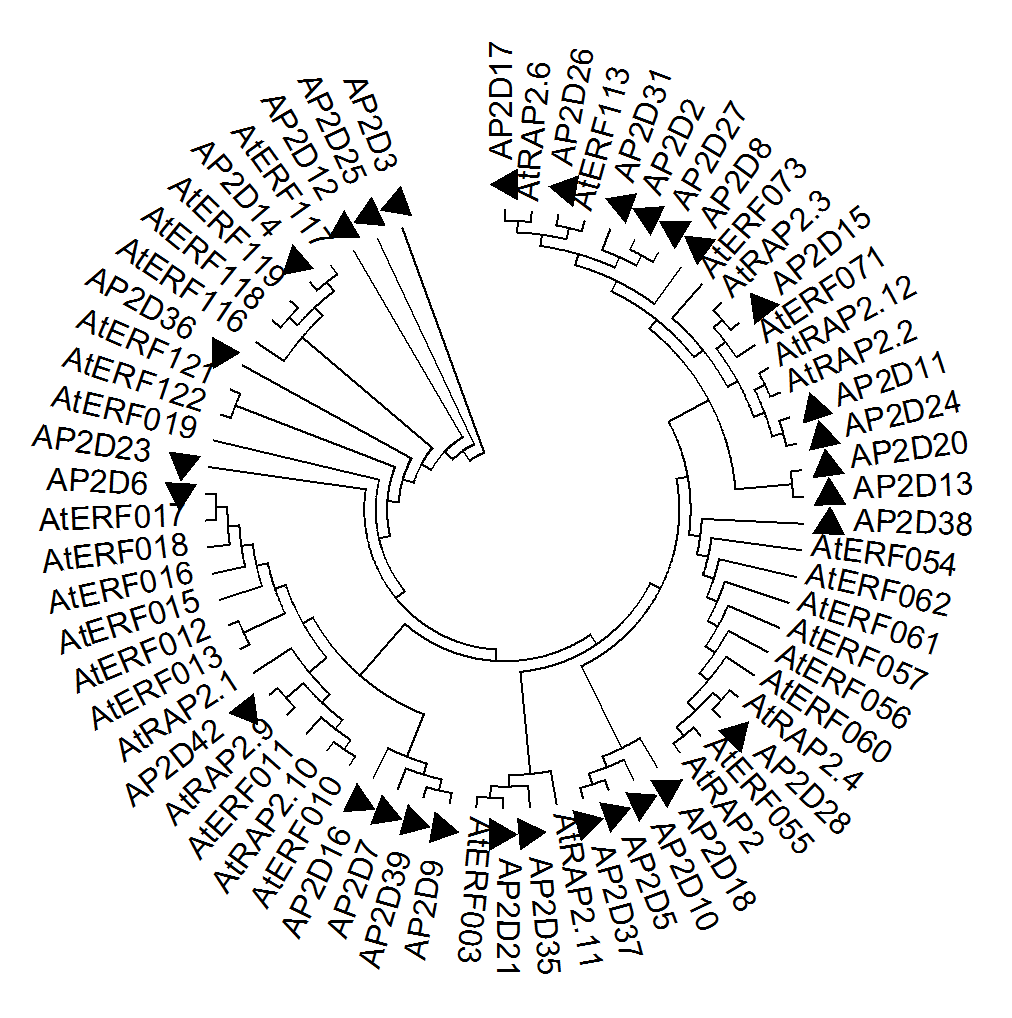

Supplement: Additional file 4: — Phylogenetic tree of AP2/ERF amino acid sequences from apple and Arabidopsis using the Neighbor-joining method in MEGA6 and reliability of tree construction estimated by boostrap method based on 1000 replicates. Apple sequences used in this analysis are indicated with black triangles. The database accession numbers of the sequences used are presented in Additional file 6. (JPEG 60 kb) [file 12870_2015_573_MOESM4_ESM.jpg]

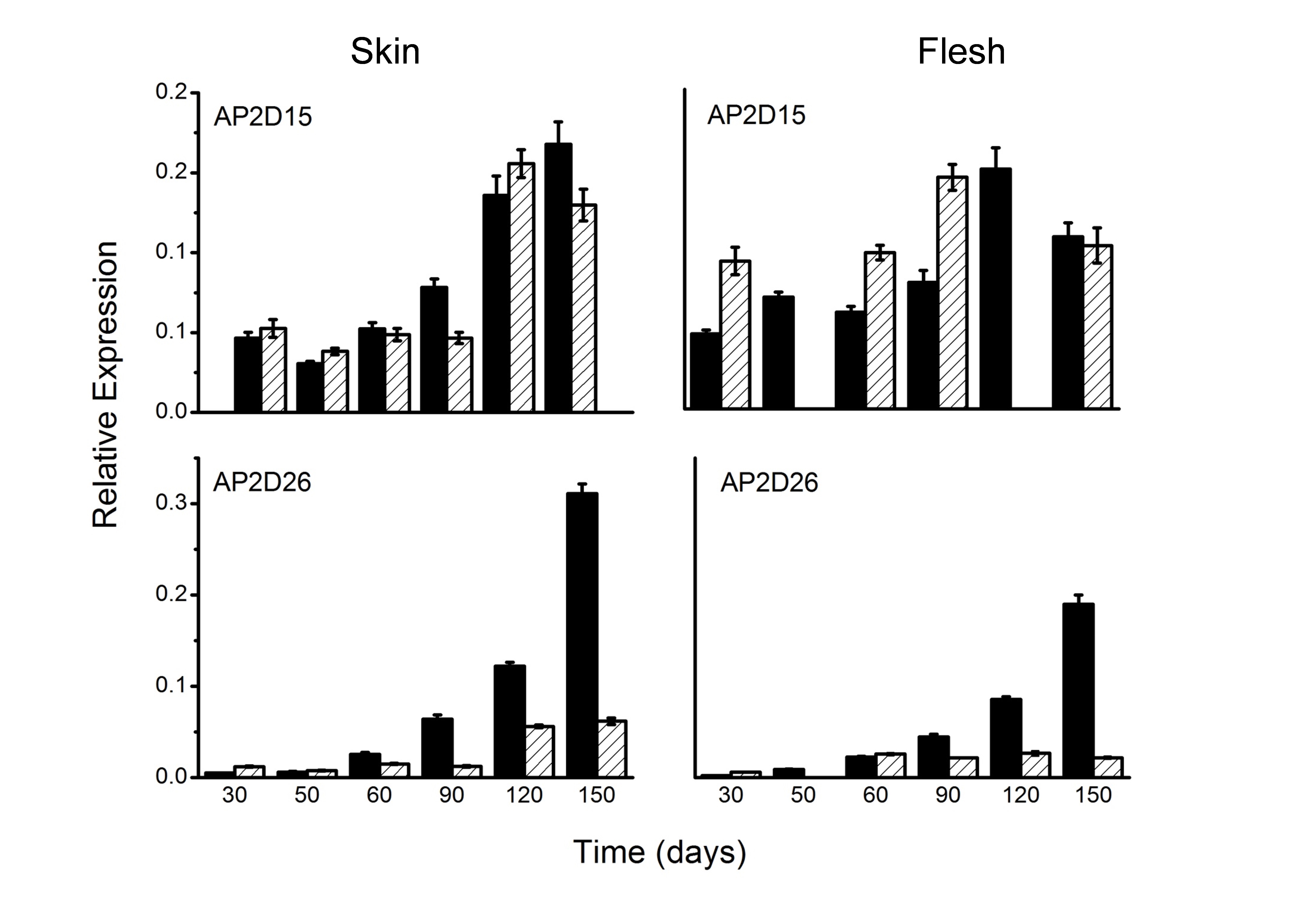

Supplement: Additional file 5: — Relative expression of AP2D15 and AP2D26 in ‘Royal Gala’ (RG) and ‘Granny Smith’ (GS) apple fruit as described in Fig. 6. Data are presented as means ± SE (n = 4). (JPEG 1012 kb) [file 12870_2015_573_MOESM5_ESM.jpg]
