# Supplementary material for: The Phytoene synthase gene family of apple (Malus x domestica) and its role in controlling fruit carotenoid content
Source: BMC Plant Biol. 2015 Jul 28;15:185. doi: 10.1186/s12870-015-0573-7 (PMC4517366; doi:10.1186/s12870-015-0573-7)
Supplement: Additional file 3: — Table of carotenoid concentration (μg/g dry weight) measured by HPLC in skin and flesh tissues of ‘Royal Gala’ and ‘Granny Smith’ apple fruit at different stages of development. (DOCX 15 kb) [file 12870_2015_573_MOESM3_ESM.docx]

Additional file 3

|  |  |  | Granny Smith | |  |  |  |  | Royal gala | |  |  |
| --- | --- | --- | --- | --- | --- | --- | --- | --- | --- | --- | --- | --- |
|  |  |  | **Fruit stage (DAFB)** | |  |  |  |  | **Fruit stage (DAFB)** | |  |  |
| Compounds | 20 | 50 | 60 | 90 | 120 | 150 | 30 | 50 | 60 | 90 | 120 | 150 |
|  |  |  |  |  |  | **Fruit skin** | |  |  |  |  |  |
| Neoxanthin/violaxanthin | 21.39 ± 0.23 | 14.56 ± 7.28 | 21.61 ± 0.41 | 22.27 ± 0.41 | n.d. | n.d. | 6.65 | n.d. | n.d. | n.d. | n.d. | n.d |
| Luteoxanthin | 23.77 ± 1.17 | 23.59 ± 0.51 | 24.25 ± 0.45 | 27.69 ± 1.53 | 23.60 ± 0.48 | 24.58 ± 0.58 | 6.97 | 22.04 ± 0.18 | 22.72 ± 0.19 | 22.75 ± 0.25 | 7.28 | n.d |
| Lutein | 62.58 ± 1.74 | 62.32 ± 1.70 | 69.62 ± 2.32 | 78.82 ± 6.03 | 44.46 ± 3.18 | 48.18 ± 0.93 | 38.53 ± 2.21 | 40.52 ± 0.88 | 36.14 ± 0.21 | 28.79 ± 0.42 | 8.04 | n.d |
| Beta-carotene | 39.72 ± 0.14 | 37.73 ± 0.59 | 41.47 ± 0.77 | 37.77 ± 1.84 | 31.16 ± 1.67 | 28.17 ± 1.67 | 27.19 ± 2.13 | 8.82 ± 8.82 | 17.89 ± 8.95 | 21.63 ± 0.40 | n.d. | n.d. |
| Total carotenoid | 147.47 ± 3.27 | 138.21 ± 7.81 | 156.95 ± 2.42 | 166.55 ± 9.61 | 99.21 ± 5.13 | 100.93 ± 3.01 | 79.34 ± 17.76 | 71.38 ± 7.88 | 76.75 ± 8.86 | 73.17 ± 0.81 | 15.31 | n.d. |
| Chlorophyll a | 42.04 ±0.98 | 43.78 ± 4.88 | 169.00 ± 18.20 | 307.10 ± 25.76 | 184.19 ± 8.02 | 203.14 ± 8.17 | 14.28 ± 14.28 | 57.41 ± 3.25 | 128.59 ± 9.57 | 94.86 ± 1.75 | 51.24 ± 6.08 | n.d |
| Chlorophyll b | 72.53 ± 3.03 | 70.67 ± 6.85 | 120.22 ± 3.82 | 141.93 ± 13.31 | 65.77 ± 7.83 | 78.78 ± 3.57 | 16.96 ± 11.95 | 51.50 ± 2.77 | 45.49 ± 0.82 | 25.03 ± 1.97 | 6.73 ± 3.38 | n.d |
| Total chlorophyll | 442.28 ± 12.38 | 425.93 ± 11.84 | 511.34 ± 7.39 | 599.96 ± 59.84 | 310.47 ± 20.31 | 328.32 ± 29.40 | 213.97 ± 16.17 | 214.95 ± 16.70 | 235.52 ± 17.83 | 119.89 ± 3.73 | 57.97 ± 8.85 | n.d |
|  |  |  |  |  |  | **Fruit flesh** | |  |  |  |  |  |
| Lutein | 15.22 ± 0.76 | 19.94 ± 0.60 | 14.14 ± 0.35 | n.d. | n.d. | n.d. | 25.01 ± 1.09 | 22.71 ± 0.26 | 21.75 ± 0.05 | 25.01 ± 1.09 | n.d. | n.d. |
| Beta-carotene | 4.39 | 12.93 ± 0.05 | n.d. | n.d. | n.d. | n.d. | 6.96 | n.d. | n.d. | n.d. | n.d. | n.d. |
| Total carotenoid | 19.60 ± 5.14 | 32.87 ± 0.60 | 14.14 ± 0.35 | n.d | n.d | n.d | 31.97± 7.83 | 22.71 ± 0.26 | 21.75 ± 0.05 | n.d. | n.d. | n.d. |
| Total chlorophyll | 205.81 ± 21.87 | 149.29 ± 6.59 | 69.40 ± 3.41 | n.d. | n.d. | n.d. | 120.01 ± 11.71 | 94.14 ± 9.38 | 71.31 ± 3.06 | n.d. | n.d. | n.d. |
