# Supplementary material for: The Phytoene synthase gene family of apple (Malus x domestica) and its role in controlling fruit carotenoid content
Source: BMC Plant Biol. 2015 Jul 28;15:185. doi: 10.1186/s12870-015-0573-7 (PMC4517366; doi:10.1186/s12870-015-0573-7)
Supplement: Additional file 6: — List of genes and associated accession numbers used in the AP2/ERFs Phylogenetic tree (Additional file 4). (DOCX 13 kb) [file 12870_2015_573_MOESM6_ESM.docx]

Additional file 6

| **Gene ID** | **Accession #** | **Gene ID** | **Accession #** |
| --- | --- | --- | --- |
| AP2D10 | MDP0000324718 | AtERF003 | AED93410 |
| AP2D11 | MDP0000308922 | AtERF010 | AED98312 |
| AP2D12 | MDP0000130802 | AtERF011 | AEE78646 |
| AP2D13 | MDP0000308379 | AtERF012 | AEE30171 |
| AP2D14 | MDP0000166779 | AtERF013 | AEE36003 |
| AP2D15 | MDP0000413387 | AtERF015 | AEE85852 |
| AP2D16 | MDP0000189347 | AtERF016 | AED92961 |
| AP2D17 | MDP0000175375 | AtERF017 | AEE29818 |
| AP2D18 | MDP0000263008 | AtERF018 | AEE35652 |
| AP2D20 | MDP0000890854 | AtERF019 | AEE30291 |
| AP2D21 | MDP0000243375 | AtERF054 | AEE85444 |
| AP2D23 | MDP0000228713 | AtERF055 | AEE31842 |
| AP2D24 | MDP0000288465 | AtERF056 | AEC07277 |
| AP2D25 | MDP0000444961 | AtERF057 | AED98006 |
| AP2D26 | MDP0000226115 | AtERF060 | AEE87118 |
| AP2D26 | MDP0000390121 | AtERF061 | AEE34234 |
| AP2D27 | MDP0000299277 | AtERF062 | AEE83304 |
| AP2D28 | MDP0000313476 | AtERF071 | AEC10854 |
| AP2D3 | MDP0000283079 | AtERF073 | AEE35312 |
| AP2D31 | MDP0000153445 | AtERF113 | AED91881 |
| AP2D35 | MDP0000172065 | AtERF116 | AEE30628 |
| AP2D36 | MDP0000128924 | AtERF117 | AEE32392 |
| AP2D37 | MDP0000839828 | AtERF118 | AEE34808 |
| AP2D38 | MDP0000165880 | AtERF119 | AEE77085 |
| AP2D39 | MDP0000148178 | AtERF121 | AED98290 |
| AP2D42 | MDP0000756341 | AtERF122 | AED98289 |
| AP2D5 | MDP0000542633 | AtRAP2 | AAP04063 |
| AP2D6 | MDP0000704216 | AtRAP2 | AEE32129 |
| AP2D7 | MDP0000198054 | AtRAP2.2 | AEE75492 |
| AP2D8 | MDP0000923579 | AtRAP2.3 | AEE75863 |
| AP2D9 | MDP0000790788 | AtRAP2.4 | AEE36065 |
|  |  | AtRAP2.6 | AEE31950 |
|  |  | AtRAP2.9 | AEE82561 |
|  |  | AtRAP20 | AEE86715 |
|  |  | AtRAP21 | AED92749 |
|  |  | AtRAP22 | AEE33023 |
